# The localization of phototropin to the plasma membrane defines a cold-sensing compartment in *Marchantia polymorpha*

**DOI:** 10.1093/pnasnexus/pgac030

**Published:** 2022-03-31

**Authors:** Satoyuki Hirano, Kotoko Sasaki, Yasuhide Osaki, Kyoka Tahara, Hitomi Takahashi, Atsushi Takemiya, Yutaka Kodama

**Affiliations:** Center for Bioscience Research and Education, Utsunomiya University, Tochigi 321-8505, Japan; Center for Bioscience Research and Education, Utsunomiya University, Tochigi 321-8505, Japan; Center for Bioscience Research and Education, Utsunomiya University, Tochigi 321-8505, Japan; Department of Biology, Graduate School of Sciences and Technology for Innovation, Yamaguchi University, 753-8512 Yamaguchi, Japan; Center for Bioscience Research and Education, Utsunomiya University, Tochigi 321-8505, Japan; Department of Biology, Graduate School of Sciences and Technology for Innovation, Yamaguchi University, 753-8512 Yamaguchi, Japan; Center for Bioscience Research and Education, Utsunomiya University, Tochigi 321-8505, Japan

**Keywords:** chloroplast movement, liverwort, phototropin, plasma membrane, thermosensor

## Abstract

Plant cells perceive cold temperatures and initiate cellular responses to protect themselves against cold stress, but which cellular compartment mediates cold sensing has been unknown. Chloroplasts change their position in response to cold to optimize photosynthesis in plants in a process triggered by the blue-light photoreceptor phototropin (phot), which thus acts as a cold-sensing molecule. However, phot in plant cells is present in multiple cellular compartments, including the plasma membrane (PM), cytosol, Golgi apparatus, and chloroplast periphery, making it unclear where phot perceives cold and activates this cold-avoidance response. Here, we produced genetically encoded and modified variants of phot that localize only to the cytosol or the PM and determined that only PM-associated phot-induced cold avoidance in the liverwort *Marchantia polymorpha*. These results indicate that the phot localized to the PM constitutes a cellular compartment for cold sensing in plants.

Significance Statement The present study identified a cellular compartment for cold sensing in plants. The blue-light photoreceptor phot acts as a cold-sensing molecule in plants. However, phot localizes in multiple cellular compartments, including the PM, cytosol, Golgi apparatus, and chloroplast periphery, preventing a clear definition of the nature of cold-sensing compartments in plant cells. In this study, we genetically regulated the subcellular localization of phot in the liverwort *Marchantia polymorpha* and found that the localization of phot to the PM defines a cold-sensing compartment in *M. polymorpha*.

## Introduction

Which cellular compartment senses cold stimulus is an interesting and important biological question. In animals, 2 members of the transient receptor potential (TRP) family of channels, TRPM8 and TRPA1, are cold sensors (1–3). Because both TRPM8 and TRPA1 localize to the plasma membrane (PM), the PM appears to define a cold-sensing compartment in animal cells. In contrast, plant genomes lack homologous genes that would encode TRP channels, suggesting that plants may have developed an independent mechanism(s) for cold sensing. We recently reported that the blue-light (BL) photoreceptor phototropin (phot), which is conserved from green algae to land plants, acts as a cold-sensing molecule in several plant species, such as the liverwort *Marchantia polymorpha*, the venus hair fern *Adiantum capillus-veneris*, and the flowering plant Arabidopsis (*Arabidopsis thaliana*) (4, 5). However, phot localizes to multiple subcellular compartments, such as the PM, the cytosol, the Golgi apparatus, and the chloroplast periphery (6–15), preventing a clear definition of the nature of any potential cold-sensing compartment in plant cells (16).

Phot possesses 2 photo-thermosensory light–oxygen–voltage (LOV1 and LOV2) domains at its N-terminus, as well as a serine/threonine kinase domain at its C-terminus (17). The LOV domains are activated by BL irradiation, leading to stimulation of the kinase domain to induce autophosphorylation and initiate signal transduction cascades that branch out to physiological responses such as changes in chloroplast positioning (17). Each LOV domain contains a key cysteine residue that forms a covalent adduct with the chromophore flavin mononucleotide (FMN) upon BL irradiation (17). This BL-induced covalent adduct exhibits thermal reversion during the photocycle (17), which has been harnessed by phot with its LOV2 domain to perceive ambient temperature and regulate intracellular chloroplast positioning (4). For example, in *M. polymorpha* grown at the normal temperature of 22°C under low-intensity BL irradiation, phot (Mpphot) adjusts the position of chloroplasts along the periclinal cell wall to maximize photosynthetic activity (accumulation response). By contrast, at the lower temperature of 5°C, Mpphot perceives the cold stimulus and induces chloroplasts to localize along the anticlinal cell wall to avoid low-intensity BL and reduce photoinhibition (the cold-avoidance response) (4). This phot-mediated cold-avoidance response has been observed in several land plants, including *M. polymorpha, A. capillus-veneris*, and Arabidopsis (4, 5).

In plant cells, phot has been reported to mainly localize to the PM in plant species including pea (*Pisum sativum*), maize (*Zea mays*), oat (*Avena sativa*), white mustard (*Sinapis alba*), morning glory (*Ipomoea nil*), *M. polymorpha*, and Arabidopsis (6–12). Because phot lacks an obvious transmembrane-spanning domain, it is believed to associate with unknown substances at the PM. In a previous study using Arabidopsis, whose genome encodes 2 phots (Atphot1 and Atphot2), as a model system, a deletion of the C-terminal 42 amino acids of Atphot2 (Atphot2-∆C42) largely abolished its ability to localize to the PM, and it instead accumulated mainly in the cytosol, suggesting that the phot C-terminal region is critical for PM localization (18). However, the identity of the critical amino acid residues involved in the PM localization of phot remained to be determined.

In our previous study of Mpphot, we documented the autophosphorylation of Mpphot under BL irradiation in plants grown at 22°C, but also observed Mpphot hyperautophosphorylation upon transfer to 5°C (4). Because Mpphot is typically largely associated with the PM, it is likely that the hyperautophosphorylated Mpphot seen at 5°C localizes to the PM. Based on this observation, we hypothesized that the localization of Mpphot to the PM, and not its accumulation in another compartment, plays a major part in cold sensing to induce the cold-avoidance response in *M. polymorpha*. Testing this hypothesis requires blocking the association of Mpphot with the PM. In this study, we established that a C-terminal di-proline motif in Mpphot strongly contributes to its PM localization. Substitution of both proline residues with alanine resulted in the accumulation of mutated Mpphot proteins only in the cytosol. In a complementary approach, we also generated an artificially PM-anchored Mpphot by genetically encoding a lipidation signal in the mutated Mpphot. Analyses of these cytosol-only and PM-only Mpphots showed that Mpphot at the PM forms a cold-sensing compartment to induce the cold-avoidance response of *M. polymorpha*.

## Results

### The Mpphot C-terminal region is important for its PM localization

**Fig. 1.**
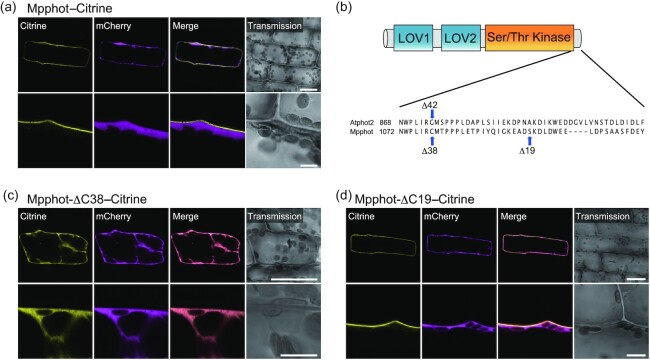
The C-terminal region of Mpphot is critical for its localization to the plasma membrane. a) Subcellular localization of Mpphot-Citrine transiently expressed in *Egeria densa*. A construct encoding Mpphot-Citrine protein was co-bombarded with a construct encoding mCherry as a cytosolic marker. b) Diagram of phototropin and the C-terminal sequences of Arabidopsis phot2 (Atphot2) and *M. polymorpha* phot (Mpphot). ∆C42 in Atphot2(18). ∆C38 and ∆C19 in Mpphot indicate C-terminal deletions. c, d) Subcellular localization of Mpphot-∆C38-Citrine (c) and Mpphot-∆C19-Citrine (d) upon transient co-bombardment with the relevant encoding constructs and *mCherry* (as cytosolic marker) in *E. densa*. a,c,d, Scale bars, 50 µm (upper panels) or 10 µm (lower panels).

To identify the residue(s) critical for Mpphot localization to the PM, we employed transient expression assays using the Brazilian waterweed (*Egeria densa*) particle bombardment system (19). *Egeria densa* is an aquatic monocot plant whose cells are easily observable by microscopy. We generated a construct encoding Mpphot fused to the Citrine yellow fluorescent protein (Mpphot–Citrine), which we transiently expressed in *E. densa* cells. Mpphot–Citrine mainly localized to the PM, but also exhibited some fluorescence in the cytosol (Fig. 1a). Importantly, cytosolic Mpphot–Citrine co-localized with the cytosolic marker mCherry (Fig. 1a).

In a previous study with Arabidopsis, a green fluorescent protein (GFP) fused to a mutated Atphot2 harboring a deletion of its C-terminal 42 amino acids (Atphot2-∆C42–GFP) accumulated mainly in the cytosol (18). A comparison of the Mpphot and Atphot2 sequences indicated that the last 38 amino acids of Mpphot correspond to the C-terminal 42 amino acids of Atphot2 (Fig. 1b). To test the contribution of the C-terminus of Mpphot, we generated and transiently expressed a new construct encoding Mpphot lacking the last 38 amino acids and fused to Citrine (Mpphot-∆C38–Citrine) in *E. densa* cells, which revealed an exclusive cytosolic localization of the fusion protein (Fig. 1c). To refine the amino acids involved in the PM localization of Mpphot, we transiently expressed a construct encoding a version of Mpphot carrying a deletion of the last 19 amino acids and fused to Citrine (Mpphot-∆C19–Citrine) in *E. densa* cells (Fig. 1b). We observed that Mpphot-∆C19–Citrine mainly localizes to the PM, as did intact Mpphot–Citrine (Fig. 1d). We concluded that the motif CMTPPPLETPIYQIGKEAD between amino acids 1,078 and 1,096 in Mpphot contains the critical amino acid residue(s) responsible for PM localization (Fig. 1b).

### A C-terminal di-proline motif in Mpphot mediates its PM localization

**Fig. 2.**
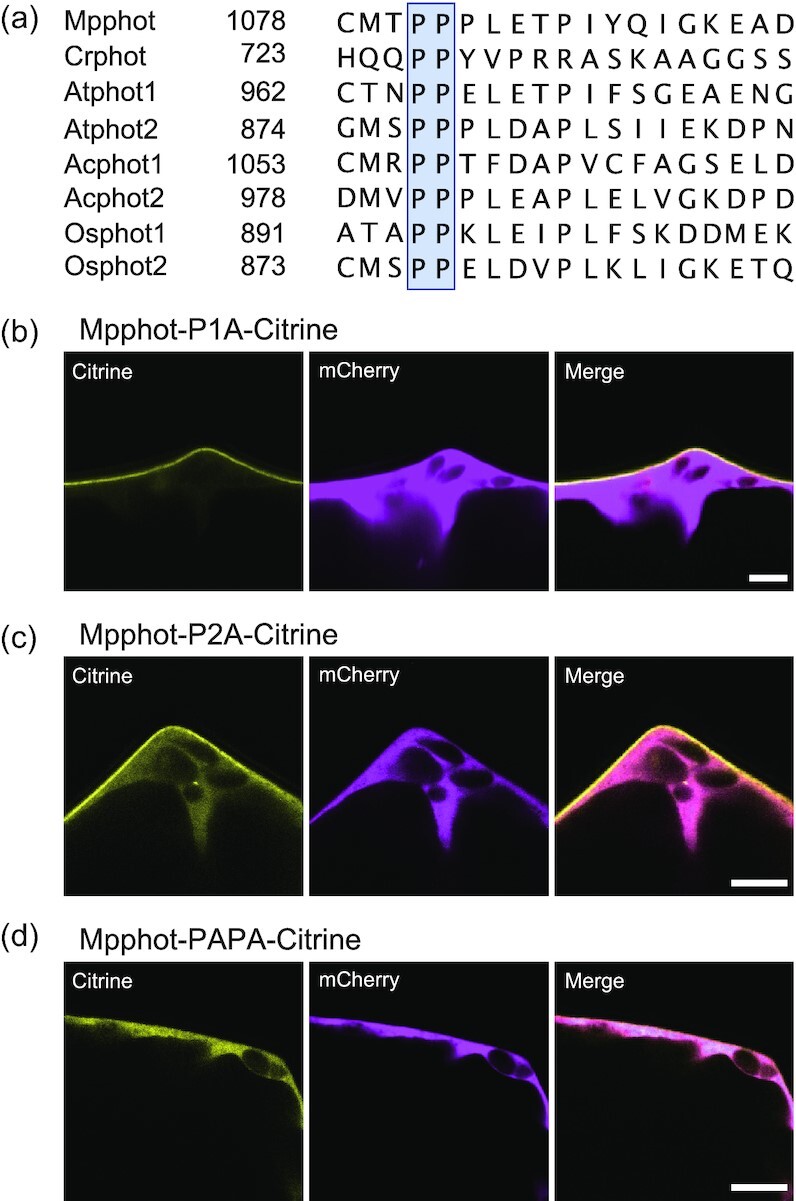
Two proline residues in the C terminus of Mpphot redundantly mediate its localization to the plasma membrane. a) Multiple sequence alignment of the C termini from various phots: Mpphot (*Marchantia polymorpha*), Crphot (*Chlamydomonas reinhardtii*), Atphot1 and Atphot2 (*Arabidopsis thaliana*), Acphot1 and Acphot2 (*Adiantum capillus-veneris*), and Osphot1 and Osphot2 (*Oryza sativa*). b–d) Subcellular localization of Mpphot-P1A-Citrine (b), Mpphot-P2A-Citrine (c), and Mpphot-PAPA-Citrine (d) following transient co-bombardment of the relevant encoding constructs with *mCherry* (as cytosolic control) in *E. densa*. Scale bars, 10 µm (b–d).

We compared amino acids 1,078–1,096 from Mpphot to the corresponding sequence of the phot proteins from various plants (*M. polymorpha*, Arabidopsis, *A. capillus-veneris*, and rice [*Oryza sativa*]) and the green alga *Chlamydomonas reinhardtii*, which revealed an absolutely conserved di-proline motif (Fig. 2a). To determine the role of this motif, we replaced each proline individually or together with alanine (P1081A [P1A], P1082A [P2A], and P1081A/P1082A [PAPA]) and transiently expressed the constructs encoding these Mpphot-P1A–Citrine and Mpphot-P2A–Citrine variants in *E. densa* cells. Similar to intact Mpphot–Citrine, Mpphot-P1A–Citrine localized strongly to the PM and accumulated only weakly in the cytosol (Fig. 2b). In contrast, Mpphot-P2A–Citrine showed equal abundance at the PM and in the cytosol (Fig. 2c). When both prolines were mutated to alanine, the resulting Mpphot-PAPA–Citrine completely lost its PM localization, accumulating only in the cytosol (Fig. 2d). The results indicate that the 2 proline residues redundantly mediate the PM localization, with P1082 contributing strongly to PM localization.

### PM localization of Mpphot in *M. polymorpha* involves the di-proline motif

**Fig. 3.**
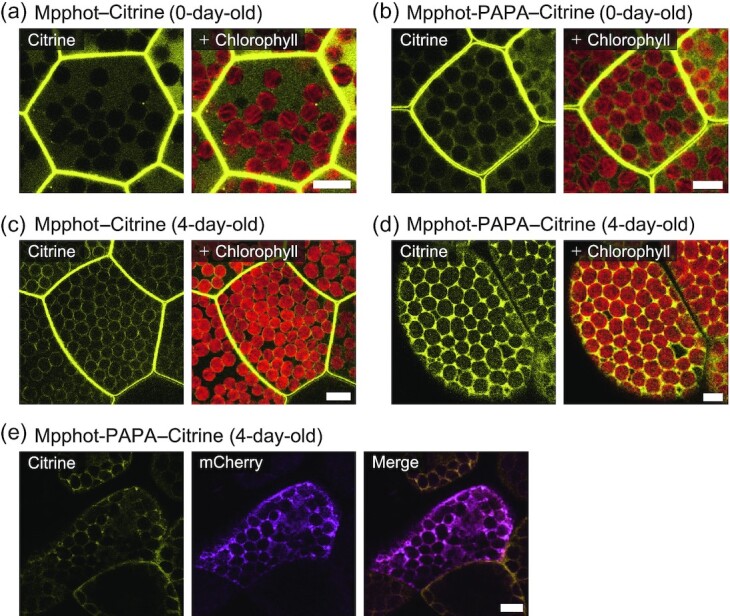
Disruption of the di-proline motif abrogates the plasma membrane localization of Mpphot in *Marchantia polymorpha*. a) Subcellular localization of Mpphot-Citrine in 0-day-old transgenic mp*phot Mpphot-Citrine* gemma cells. b) Subcellular localization of Mpphot-PAPA-Citrine in 0-day-old transgenic mp*phot MpPHOT-PAPA-Citrine* #3 gemma cells (PAPA line #3). c) Subcellular localization of Mpphot-Citrine in 4-day-old transgenic gemmaling cells (mp*phot MpPHOT-Citrine*). d) Subcellular localization of Mpphot-PAPA-Citrine in 4-day-old transgenic gemmaling cells (mp*phot MpPHOT-PAPA-Citrine* #3: PAPA line #3). e) Co-localization of Mpphot-PAPA-Citrine and cytosolic mCherry in 4-day-old transgenic gemmaling cells (PAPA line #3). The construct encoding mCherry was transiently expressed via particle bombardment. All experiments were performed at 22°C (a–e). Scale bars, 10 µm (a–e).

We tested the effect of the di-proline motif on the localization pattern of Mpphot in *M. polymorpha* cells by generating stable transformants in mp*phot* knockout mutant cells (12) through Agrobacterium (*Agrobacterium tumefaciens*)-mediated gemma transformation (20, 21). Accordingly, we obtained 3 independent lines accumulating Mpphot-PAPA–Citrine (mp*phot MpPHOT-PAPA–Citrine* lines #3, #5, and #7: abbreviated as PAPA lines #3, #5, and #7). As a control, we also transformed the mp*phot* mutant with *MpPHOT–Citrine* (mp*phot MpPHOT–Citrine*) for functional complementation (15, 22). We observed that Mpphot-PAPA–Citrine is unexpectedly present at the PM and the cytosol in 0-day-old transgenic gemma cells from PAPA line #3, similar to intact Mpphot–Citrine (Fig. 3a and b). We cultured transgenic gemmae for 4 days (such a cultured gemma is called a gemmaling) at 22°C in continuous white fluorescent light; Mpphot–Citrine remained at the PM and appeared to decrease in abundance in the cytosol, accumulating instead around chloroplasts (Fig. 3c) as previously reported (15). In sharp contrast, Mpphot-PAPA–Citrine localized only in the cytosol under the same growth conditions (Fig. 3d). We confirmed the cytosolic localization of Mpphot-PAPA–Citrine by transiently expressing a construct encoding the red fluorescent protein mCherry as a cytosolic marker in 4-day-old transgenic PAPA line #3 gemmaling cells (Fig. 3e). These results indicate that the di-proline motif of Mpphot mediates its PM localization in cultured gemmaling cells, but only in 4-day-old gemmaling cells and not in 0-day-old gemma cells. Furthermore, the PM localization of Mpphot-PAPA–Citrine was completely disrupted, as Mpphot-PAPA–Citrine only localized to the cytosol (Fig. 3d and e).

### Mpphot at the PM perceives cold temperatures

**Fig. 4.**
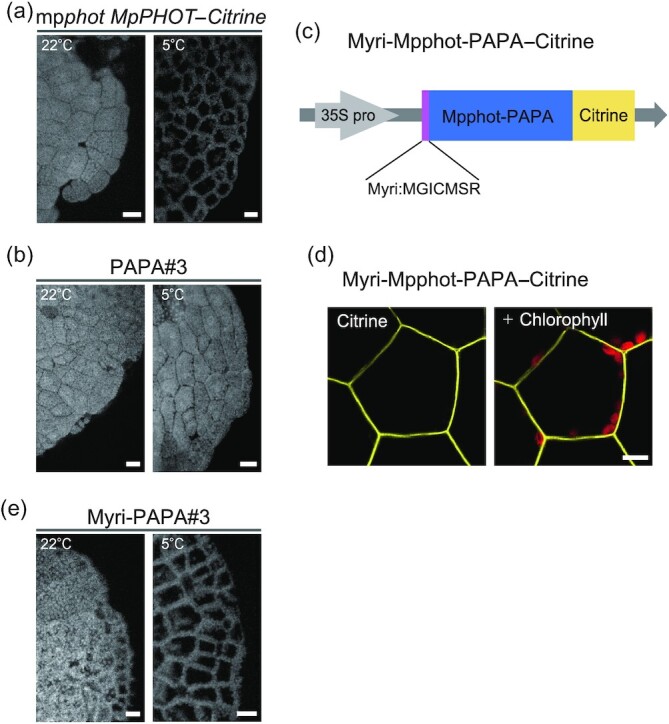
Mpphot at the plasma membrane senses cold signals. a) The cold-avoidance response is induced by Mpphot-Citrine. The accumulation and cold-avoidance responses were observed at 22°C and 5°C, respectively, in 4-day-old transgenic mp*phot MpPHOT-Citrine* gemmaling cells. b) No cold-avoidance response is induced by Mpphot-PAPA-Citrine. The accumulation response was observed in 4-day-old transgenic mp*phot MpPHOT-PAPA-Citrine* #3 gemmaling cells (PAPA line #3) at both 22°C and 5°C. Note that the Citrine fluorescence intensity in the cytosol of PAPA line #3 is higher than that of mp*phot MpPHOT-Citrine* transgenic cells (Figure S1, Supplementary Material). c) Diagram of Myri-Mpphot-PAPA-Citrine. The myristoylation signal sequence (Myri: MGICMSR)(23) was fused to the N terminus of Mpphot-PAPA-Citrine. The coding sequence of *Myri-MpPHOT-PAPA-Citrine* was driven by the cauliflower mosaic virus 35S promoter (35S pro). d) Subcellular localization of Myri-Mpphot-PAPA-Citrine in 4-day-old transgenic mp*phot Myri-MpPHOT-PAPA-Citrine* #3 gemma cells (Myri-PAPA line #3). e) The cold-avoidance response is induced by Myri-Mpphot-PAPA-Citrine. The accumulation and cold-avoidance responses were observed at 22°C and 5°C, respectively, in 4-day-old transgenic gemmaling cells of Myri-PAPA line #3. Note that Citrine fluorescence intensity at the plasma membrane of Myri-PAPA line #3 is higher than that of mp*phot MpPHOT-Citrine* (Figure S3, Supplementary Material). Scale bars, 50 µm (a, b, e) or 10 µm (d).

To investigate whether cytosolic Mpphot-PAPA–Citrine contributes to cold sensing in the cell, we analyzed the cold-avoidance response of 4-day-old gemmaling cells from PAPA lines. Based on Citrine fluorescence intensity, cytosolic Mpphot-PAPA–Citrine in all PAPA lines accumulated to higher levels than cytosolic Mpphot–Citrine in the mp*phot MpPHOT–Citrine* lines (Figure S1, Supplementary Material). We induced a cold-avoidance response by incubating 4-day-old cells at 5°C under low-intensity BL for 24 h. Chloroplasts localized along the anticlinal cell wall in mp*phot* *MpPHOT–Citrine* gemmaling cells, thus exhibiting a typical cold-avoidance response (Fig. 4a and Figure S1, Supplementary Material). By contrast, chloroplasts remained along the periclinal cell wall in PAPA lines (cytosol-type Mpphot), signifying a loss of cold-avoidance response (Fig. 4b and Figure S1, Supplementary Material). Note that the subcellular localization patterns of Mpphot–Citrine and Mpphot-PAPA–Citrine were not changed at 5°C under low-intensity BL for 24 h (Figure S2, Supplementary Material). These results, therefore, suggested that Mpphot at the PM contributes to cold sensing in *M. polymorpha* cells.

To further test whether the pool of Mpphot at the PM senses cold, we artificially tethered Mpphot-PAPA–Citrine to the PM by adding a myristoylation signal sequence (Myri) (23) to the N-terminus of Mpphot-PAPA–Citrine (Myri-Mpphot-PAPA–Citrine) (Fig. 4c), and introducing the resulting construct into mp*phot* knockout mutant cells (mp*phot Myri*-*MpPHOT-PAPA–Citrine* lines #1–3: abbreviated Myri-PAPA#1–3). We measured the abundance of Myri-Mpphot-PAPA–Citrine at the PM based on Citrine fluorescence intensity (Figure S3, Supplementary Material). Citrine levels at the PM were comparable between the Myri-PAPA#1 line and the control mp*phot* *MpPHOT–Citrine* line, while Myri-PAPA lines #2 and #3 showed approximately 2-fold higher Citrine fluorescence than the same mp*phot* *MpPHOT–Citrine* line (Figure S3, Supplementary Material). In addition, we observed Citrine fluorescence only at the PM of 4-day-old gemmaling cells from Myri-PAPA line #3, but not in the cytosol or the chloroplast periphery (Fig. 4d). Exposure of 4-day-old gemmaling cells to 5°C under low-intensity BL for 24 h induced a cold-avoidance response in Myri-PAPA lines #2 and #3, but not in Myri-PAPA line #1 (Fig. 4e and Figure S3, Supplementary Material), indicating that cold sensing in *M. polymorpha* requires high Myri-Mpphot-PAPA–Citrine abundance. Importantly, the opposite results obtained with Mpphot-PAPA–Citrine (cytosol localization) and Myri-Mpphot-PAPA–Citrine (PM localization) indicate that the localization of Mpphot to the PM is a prerequisite for cold-sensing and the cold-avoidance response in *M. polymorpha*.

## Discussion

This study identified a di-proline motif (PP motif) in Mpphot as being essential for its PM localization and showed that Mpphot at the PM senses cold to induce the cold-avoidance response in *M. polymorpha*. Our findings reveal that the localization of phot at the PM defines a cold-sensing compartment in plants.

The localization to the PM is a conserved property of all phot proteins from various plant species (6–12), but the critical residues responsible for this pattern were unknown until now. Here, we identified 2 proline residues within Mpphot as redundantly mediating the PM localization. That Mpphot retained at least a partial PM localization when either proline was replaced by alanine, and only their simultaneous substitution completely abolished the localization to the PM, indicates that these 2 proline residues endow Mpphot with a higher tolerance to natural variation while maintaining phot at the PM.

Phot belongs to the extended AGC (cAMP-dependent protein kinase, cGMP-dependent protein kinase, and protein kinase C) kinase family and more specifically within the plant-specific AGC VIII subfamily (24). The Arabidopsis genome encodes 23 AGCVIII kinases that can be further separated into 4 subgroups (AGC1, AGC2, AGC3, and AGC4) (25). The *M. polymorpha* genome harbors only 4 genes for AGCVIII kinases, which can be assigned to 3 subgroups based on sequence similarity: AGC1 (Mpzg01290 and Mp8g16840), AGC2 (Mp6g08530), and AGC4 (Mp5g03810, Mpphot). Because of its lower apparent degree of redundancy, *M. polymorpha* may be more suitable than Arabidopsis as a model in which to analyze the function of AGCVIII kinases. AGC kinase members both within and outside the AGCVIII subfamily share sequences similar to the phot C termini, with 1 or more proline residues (Figure S4a, Supplementary Material). For instance, animal AGC kinase Akt proteins possess a conserved PPxxP motif similar to the phot di-proline motif that was reported to be involved in an interaction with the SH3 (SRC homology 3) domain of the tyrosine kinase Src (26) (Figure S4b, Supplementary Material). Although the PxxP motif is conserved in *C. reinhardtii* phot as a PPxxP sequence, the second proline of the PxxP motif is replaced by D (aspartic acid) or E (glutamic acid) in the phot from *M. polymorpha*, Arabidopsis, *A. capillus-veneris*, and rice, although all plant phot proteins are associated with the PM (Fig. 2a and Figure S4b, Supplementary Material). Thus, the PP motif, not the PxxP motif, mediates the PM localization of plant phot. We hypothesize that other plant AGC kinases with a di-proline motif may also localize to the PM. More broadly, the di-proline motif may constitute a new PM localization signal in various organisms.

We showed that Mpphot-PAPA–Citrine localizes to the PM and the cytosol in 0-day-old gemma cells, but relocalized solely to the cytosol in 4-day-old gemmaling cells (Fig. 3). Because 0-day-old gemma cells are static, dormant cells that are shaded inside the gemma cup, we speculate that Mpphot-PAPA lacking the C-terminal di-proline motif can associate with the PM under these static conditions, but that the di-proline motif is required for phot to maintain its PM localization in nonstatic conditions such as those in cultured cells. A fusion between GFP and Arabidopsis phot2 lacking the last 42 amino acids (Atphot2–∆C42) was reported to be cytosolic, although biochemical fractionation analyses also detected Atphot2–∆C42 in the insoluble fraction (18). We obtained similar results when conducting fractionation of 2-week-old transgenic gemmaling cells from PAPA line #3 (Figure S5, Supplementary Material). We speculate that the protocol followed for biochemical fractionation involving mashing and mixing is similar to the static conditions of 0-day-old gemma cells. Taking these results together, we conclude that Mpphot-PAPA likely interacts with an unknown substance(s) at the PM and that an unknown mechanism(s) other than the di-proline motif allows phot to localize to the PM under static conditions.

In wild-type Arabidopsis seedlings, Atphot1 localizes to the PM, in the cytosol and at the chloroplast envelope, and mediates only the accumulation response; Atphot2 localizes to the PM, the cytosol, the Golgi apparatus, and the chloroplast envelope and mediates the accumulation, avoidance, and cold-avoidance responses (9, 13, 14, 27–29). The artificial tethering of Atphot1–GFP (or Citrine) and Atphot2–GFP to the PM was accomplished by adding an N-terminal myristoylation (Myri) or C-terminal farnesylation (Farn) signal to Atphot1 (Myri-Atphot1–Citrine and Atphot1–GFP-farn) and Atphot2 (Myri-Atphot2–GFP) (30, 31). Both myristoylation and farnesylation of Atphot blocked its localization to other compartments. In addition, PM-tethered Atphot1 and Atphot2 induced an accumulation response, whereas Myri-Atphot2–GFP induced the avoidance response (30, 31). These results indicated that the PM localization of Atphot1 and Atphot2 is sufficient to initiate the accumulation and/or avoidance responses under normal ambient temperature conditions. In this study, we added an N-terminal myristoylation sequence to Mpphot-PAPA–Citrine; the resulting Myri-Mpphot-PAPA–Citrine successfully induced not only the cold-avoidance response (Fig. 4e and Figure S3, Supplementary Material) but also the accumulation and avoidance responses (Figure S6, Supplementary Material), suggesting that the di-proline motif does not affect the biochemical function of Mpphot. Anyway, the cold-avoidance response could be induced by the cold-sensor Mpphot at the PM. In our previous study, we reported that the cold-avoidance response is dependent on actin filaments in *M. polymorpha* (32), and found that long filamentous actin structures along the periclinal cell walls relocated together with chloroplasts during the cold-avoidance response (32). In the vicinity of the PM attached to the periclinal cell walls, the cold-sensor Mpphot may transduce the signal to reorganize the filamentous actin structures.

Although the N-terminal myristoylation of Mpphot-PAPA mimicked the PM localization of Mpphot, the high abundance of Myri-Mpphot-PAPA–Citrine was necessary to induce the cold-avoidance response (Fig. 4 and Figure S3, Supplementary Material). We considered 2 possible explanations for this observation. First, the N-terminal myristoylation signal may not fully substitute for the mechanism by which Mpphot normally localizes to the PM. Indeed, the C-terminal di-proline motif of Mpphot mediates its PM localization, suggesting that Mpphot may tether to the PM *via* its C-terminus. By contrast, the myristoylation signal will tether Mpphot-PAPA–Citrine to the PM *via* its N-terminus, which may not be sufficient. Second, the subcellular localization of Mpphot to compartments other than the PM may be required. Mpphot localizes not only at the PM, but also to the cytosol and the chloroplast periphery (12, 15, 22). We showed previously that chloroplast relocation movement, including that induced by the cold-avoidance response, takes place when Mpphot increases its association with the chloroplast peripheral residency while decreasing its cytosolic residency in *M. polymorpha* (15). Furthermore, a version of Atphot2 artificially localizing to the chloroplast envelope partially induced the avoidance response to strong light (31). In this context, Mpphot at the chloroplast periphery may support the cold-avoidance response and/or avoidance response induced by PM-tethered Mpphot. An exploration of these 2 possibilities will constitute the basis for future work.

Importantly, this study differs from previous PM tethering attempts with Atphot1 and Atphot2 (30, 31), which used the wild-type sequences, in the use of cytosolic Mpphot-PAPA as the template for the genetically encoded PM localization. Although the lipidation of Atphot1 and Atphot2 appeared to block their accumulation in other compartments, the ability of these proteins to localize to the PM was thought to have been retained. In contrast, Mpphot-PAPA–Citrine, used here, had lost the potential to associate with the PM on its own in cultured gemmaling cells, making it an ideal template for myristoylation through the engineering of Myri-Mpphot-PAPA–Citrine. Although the mechanism underlying the normal PM localization pattern of wild-type phot is still unknown, the artificial myristoylation signal, almost, fully compensated for the loss of the di-proline motif and induced the accumulation, avoidance, and cold-avoidance responses (Fig. 4 and Figure S6, Supplementary Material). Future comparative analysis of PM localization systems using cytosolic Mpphot-PAPA may illuminate the exact nature of the phot PM localization mechanism.

In this study, we concluded that Mpphot localizing at the PM senses the cold stimulus and induces the cold-avoidance response in *M. polymorpha*, thus defining the phot-localized PM as a cold-sensing compartment in plant cells. Recently, several molecules with temperature-sensing properties have been discovered in plants: for example, phytochrome, EARLY FLOWERING3 (ELF3). and the transcripts of *PHYTOCHROME INTERACTING FACTOR7* (*PIF7*) to perceive warm temperatures (e.g. 22°C vs. 27°C) and phot for cold sensing (e.g. 22°C vs. 5°C) (4, 16, 33–37). Of these molecules, only phot localizes to the PM, as animal TRPs do (9, 12, 14–16), although their PM localization mechanisms are likely distinct. Although different cold-sensing molecules may have independently evolved in plants and animals, the PM localization of cold-sensing molecules appears to be a common feature, underscoring a potential common mechanism.

## Methods

### Plant materials and culture conditions

*Egeria densa* was purchased from Kanseki Co. Ltd. (Utsunomiya, Japan) and maintained in fresh water under approximately 10 µmol photons m^–2^ s^–1^ continuous white fluorescent light (FL40SW, NEC Corporation, Tokyo, Japan) in a culture room at 22°C before use for particle bombardment (19). *Marchantia polymorpha* was asexually maintained on half-strength B5 medium with 1% (w/v) agar (BOP, SSK Sales Co., Ltd., Shizuoka, Japan) under approximately 70 µmol photons m^–2^ s^–1^ continuous white fluorescent light in the culture room at 22°C (38). The light spectrum of the white fluorescent light was previously reported (39). The male accession Takaragaike-1 (Tak-1) was used as the wild type. The mp*phot* knockout mutant line was kindly provided by Professor Takayuki Kohchi (Kyoto University) (12) and was used as the material to produce mp*phot MpPHOT–Citrine*, mp*phot* *MpPHOT-PAPA–Citrine* (PAPA lines #3, #5, and #7), and mp*phot Myri-MpPHOT-PAPA–Citrine* (Myri-PAPA lines #1–3) as described below. mp*phot MpPHOT–Citrine* was previously reported (15).

### Plasmid construction

To construct plasmids, Gateway Cloning Technology (Invitrogen) was used according to the manufacturer's instructions. Template plasmid DNA, primers, and the resulting plasmids are listed in Table S1 (Supplementary Material). The DNA fragments encoding Mpphot∆C19 and Mpphot∆C38 were amplified by PCR and cloned into the pDONR207 vector *via* BP Gateway reaction to produce pDONR207-MpPHOT∆C19 and pDONR207-MpPHOT∆C38. Substitutions of proline(s) within Mpphot (Mpphot-P1A, Mpphot-P2A, and Mpphot-PAPA) were performed by *Dpn*I-mediated site-directed mutagenesis (40). For single substitutions (Mpphot-P1A and Mpphot-P2A), pDONR207-MpPHOT (22) was used as a template for mutagenesis to yield pDONR207-MpPHOT-P1A and pDONR207-MpPHOT-P2A. pDONR207-MpPHOT-P1A was subsequently used as a template for the second substitution to produce pDONR207-Mpphot-PAPA. To add a myristoylation signal sequence (Myri: MGICMSR) (23) to the N-terminus of Mpphot-PAPA, a primer containing a 21-mer sequence (5′-ATGGGAATCTGCATGTCCCGC-3′) encoding Myri was designed and used to amplify the DNA fragment encoding Myri-Mpphot-PAPA, which was cloned into the pDONRZeo vector by the BP reaction to produce pDONRZeo-Myri-Mpphot-PAPA. All plasmids in the pDONR207 or pDONRZeo backbones were recombined with pMpGWB306, a destination vector for C-terminal yellow fluorescent protein (Citrine)-tag fusions and expression in plants (41), *via* LR reaction to generate the binary vectors pMpGWB306-MpPHOT-P1A–Citrine, pMpGWB306-MpPHOT-P2A–Citrine, pMpGWB306-Mpphot-PAPA–Citrine, and pMpGWB306-Myri-Mpphot-PAPA–Citrine. Note that pMpGWB306 contains a resistance gene for the herbicide chlorsulfuron. For transient expression of *mCherry* in *E. densa* and *M. polymorpha*, pDONR207-mCherry (19) was recombined with the destination vector pGWT35S (42) *via* LR reaction to generate pGWT35S-mCherry.

### Transient expression in *E. densa* and *M. polymorpha via* particle bombardment

Particle bombardment in *E. densa* and *M. polymorpha* was performed as previously reported (19, 43). The binary vector (1 µg µL^−1^) encoding wild-type or mutant versions of Mpphot with a C-terminal Citrine tag was mixed with pGWT35S-mCherry (1 µg µL^−1^) as a cytosolic control.

### Stable transformation of *M. polymorpha*

The binary vectors were transformed into Agrobacterium (*A. tumefaciens*) strain GV2260. Subsequently, mp*phot* gemmae were transformed *via* Agrobacterium-mediated transformation by the G-AgarTrap method (20, 21, 44). The resulting primary (T_1_) transformants were selected under the herbicide chlorsulfuron and cultivated for 1 month. Transgenic G_1_ gemmae were obtained from the gemma cups of T_1_ thalli; transgenic G_2_ gemmae were used for all experiments. The transformants produced in this study were designated mp*phot* *MpPHOT-PAPA–Citrine* (PAPA lines #3, #5, and #7) and mp*phot* *Myri-MpPHOT-PAPA–Citrine* (Myri-PAPA lines #1–3).

### Fluorescence imaging analysis

Analyses of Citrine and chlorophyll fluorescence in transformed cells of *E. densa* and *M. polymorpha* were performed according to our previous studies (15, 19, 22). Briefly, Citrine and chlorophyll fluorescences were observed by confocal laser scanning microscopy (SP8X system, Leica Microsystems, Wetzlar, Germany). To clearly observe Citrine fluorescence, the time-gated method (22) was used to block chlorophyll fluorescence.

To quantify Citrine fluorescence intensity in the cytosol and at PM regions, Citrine images were taken at a resolution of 512 × 512 pixels (Figures S1 and S3, Supplementary Material). For each region, the fluorescence intensities were randomly measured for 100 positions on the single cell image (total 100 pixels), and an averaged intensity was calculated. The measurements and calculation were performed with 3 cells (*n* = 3), and statical analysis was performed using GraphPad Prism (GraphPad Software, CA).

### Analysis of chloroplast relocation movement

To analyze accumulation and avoidance responses, a temperature-regulated microscope with a BL microbeam system was used (4, 45). To observe the accumulation response, gemmae were cultured at 22°C under continuous white fluorescent light (70 µmol photons m^–2^ s^–1^) for 3 days and incubated in the dark for 1 day (24 h) to induce the dark positioning response, during which chloroplasts localize along the anticlinal wall with neighboring cells (12). To observe the avoidance response, gemmae were cultured at 22°C under continuous white fluorescent light (70 µmol photons m^–2^ s^–1^) for 4 days. To induce the accumulation and avoidance responses under the microscope, the BL microbeam was used at a setting of 10 W m^–2^ (approximately 30 µmol photons m^–2^ s^–1^) or 100 W m^–2^ (approximately 430 µmol photons m^–2^ s^–1^), respectively. The light intensity was measured with a power meter (1918-R; Newport Corporation, CA) with a silicon detector (918D-SL-OD1; detector active area: 1 cm^2^; Newport Corporation). The cell temperature was kept at 22°C, and chloroplast images were taken every 1 min for 90 min long. To quantify chloroplast relocation movement from the acquired images, the migration distance of 5 randomly selected chloroplasts was measured in ImageJ, using the distance between the center of the chloroplast and the center of the microbeam. The distance between the chloroplast and the microbeam before microbeam irradiation was set to 0, and the change in distance after irradiation was determined.

To analyze the cold-avoidance response, temperature-controlled incubators (IJ100 and IJ101, Yamato Scientific Co., Ltd, Japan) equipped with light‐emitting diodes (LEDs) for BL (450 nm, ISL-150 × 150-BB45, CCS Inc) were used. The light intensity was measured with a light meter (LI-250A; LI-COR Bioscience). To observe the cold-avoidance response, gemmae were cultured at 22°C under continuous white fluorescent light (70 µmol photons m^–2^ s^–1^) for 3 days and incubated at 22°C under weak BL (25 µmol photons m^–2^ s^–1^) for 1 days (24 h) to adapt to the new condition and induce the accumulation response. The adapted gemmae were transferred to 5°C for 24 h under the same BL intensity to induce the cold-avoidance response.

### Cellular fractionation and immunoblot analysis

For cellular fractionation, 2-week-old gemmalings were mixed in fractionation buffer (50 mM MOPS-KOH pH 7.5, 2.5 mM EDTA, 100 mM NaCl, 0.5 mM phenylmethylsulfonyl fluoride [PMSF], 10 µM leupeptin, 2 mM DTT, 10 mM NaF, 0.5 mM ammonium molybdate, and 100 nM calyculin A). The homogenate was subjected to centrifugation at 10,000 *g* for 10 min at 4°C (MX-300, Tomy Seiko Co., Ltd., Tokyo, Japan), and the supernatant was obtained as the total fraction. The protein concentrations of the supernatant were quantified using a Pierce 660 nm Protein Assay Regent (Thermo Fisher Scientific). To divide the total fraction into soluble and insoluble fractions, the supernatant containing 60 µg of protein was subjected to ultracentrifugation at 100,000 *g* for 60 min at 4°C using a rotor (TLA 110; Optima MAX-E ultracentrifuge, Beckman Coulter).

The protein extracts of the total (60 µg), soluble (split from the total), and insoluble (split from the total) fractions were mixed with trichloroacetic acid to a final concentration of 10% [w/v] and incubated on ice for 10 min (46). After centrifugation at 17,800 *g* for 10 min at 4°C, the pellets were washed with 50 mM Tris-HCl, pH 8.0, solubilized with sample buffer (0.825% [w/v] SDS, 8.25% [w/v] sucrose, 0.825 mM EDTA, 0.003% [w/v] Coomassie brilliant blue, 4.17% [v/v] 2-mercaptoethanol, and 8.25 mM Tris-HCl, pH 8.0), and incubated at 95°C for 3 min. Proteins were subjected to SDS-PAGE on an 7.5% (w/v) polyacrylamide gel and transferred to a nitrocellulose membrane. Mpphot was detected with anti-Mpphot antibody, as previously reported (4).

## Authors' Contributions

Y.K. conceived and designed the study. H.S., K.S., Y.O. performed most of the experiments. K.T. and A.T. carried out biochemical experiments. A.T. and Y.K. supervised the experiments. Y.K. wrote the manuscript with contributions from all authors. S.H. prepared the figures.

## Supplementary Material

pgac030_Supplemental_Materials_v2

## Data Availability

The data supporting the findings of this study are available within the article and its supplementary material.
