# Supplementary material for: The localization of phototropin to the plasma membrane defines a cold-sensing compartment in Marchantia polymorpha
Source: PNAS Nexus. 2022 Mar 31;1(2):pgac030. doi: 10.1093/pnasnexus/pgac030 (PMC9802274; doi:10.1093/pnasnexus/pgac030)
Supplement: pgac030_Supplemental_Materials_v2 [file pgac030_supplemental_materials_v2.pdf]

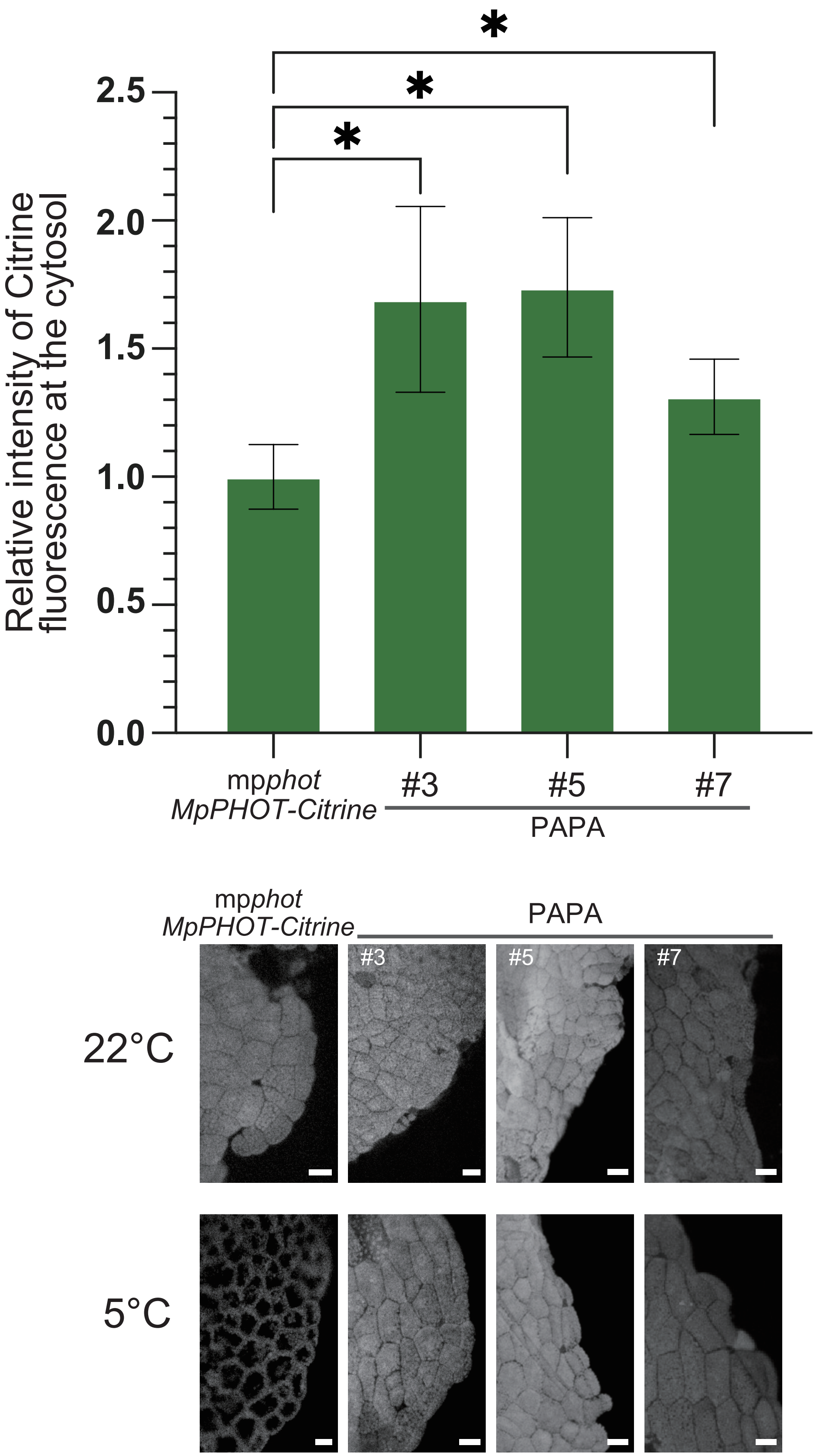

Figure S1. Comparison of fluorescence intensity for Citrine fusion proteins in the cytosol. a, Relative fluorescence intensity of Citrine fusion proteins in the cytosol, shown as the average from one hundred regions per cell ($n = 3$). Asterisk indicates statistical significance from unpaired Student's *t*-test ($P < 0.05$). **b**, No cold-avoidance response is induced in mp*phot MpPHOT-PAPA-Citrine* (PAPA lines # 3, #5, and #7). Images for mp*phot MpPHOT-PAPA-Citrine* and PAPA line #3 are the same as in Fig. 4a and 4b. Scale bars, 50 µm.

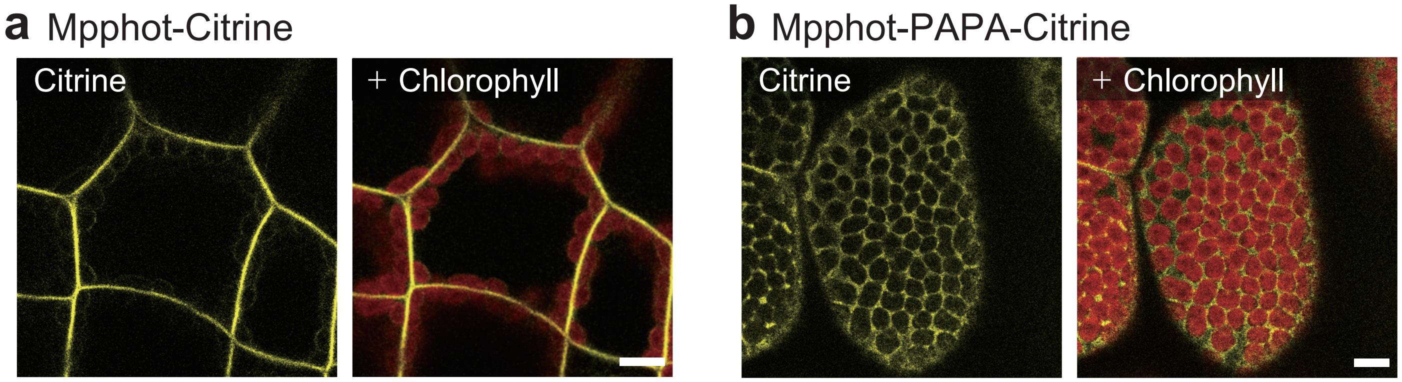

Figure S2. Subcellular localization of Mpphot-Citrine and Mpphot-PAPA-Citrine at 5° C. Subcellular localization of Mpphot-Citrine in 4-day-old transgenic mp*phot Mpphot-Citrine* gemma cells at 5° C. **b**, Subcellular localization of Mpphot-PAPA-Citrine in 4-day-old transgenic mp*phot MpPHOT-PAPA-Citrine* #3 gemma cells (PAPA line #3) at 5° C. Scale bars, 10 μm.

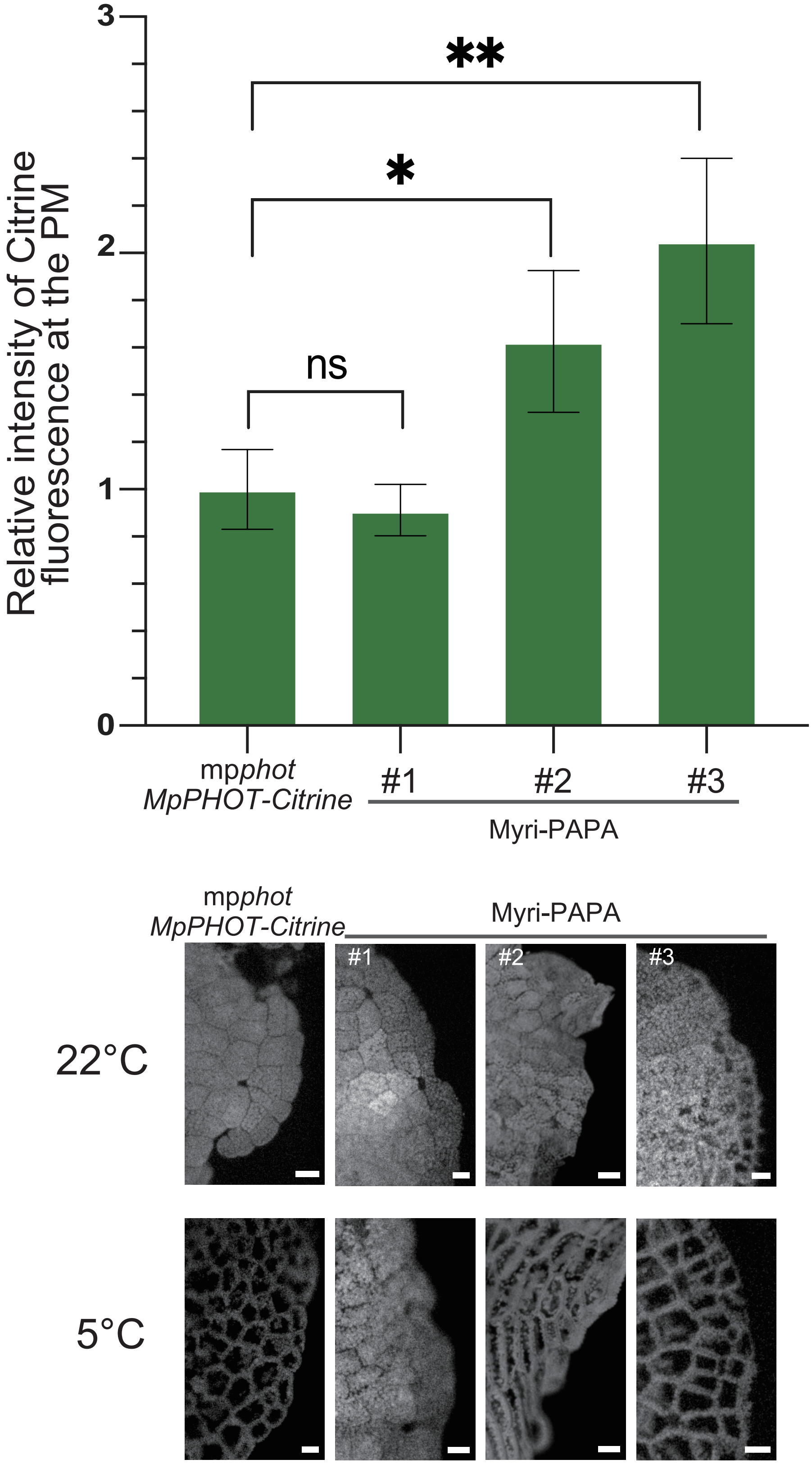

Figure S3. Comparison of fluorescence intensity for Citrine fusion proteins at the plasma membrane. a, Relative fluorescence intensity of Citrine fusion proteins at the plasma membrane, shown as the average from 100 regions per cell (n = 3). Asterisks indicate statistical significance from unpaired Student's t-test (*P < 0.05, **P < 0.01). ns, no significant difference. **b**, The cold-avoidance response is induced in mp*phot Myri-MpPHOT-PAPA-Citrine* (Myri-PAPA lines #1, #2, and #3). Images for mp*phot MpPHOT-PAPA-Citrine* and Myri-PAPA line #3 are the same as in Fig. 4a and 4e. Scale bars, 50 μm.

a

A.thaliana

At3g44610	AGC1-12	426	-PPFLP--
At4g26610	AGC1-2/D6PKL1	469	-PPEIP--
At5g55910	AGC1-1/D6PK	459	-PPEIP--
At5g47750	PK5/D6PKL2	540	-PPEIP--
At3g27580	PK7/D6PKL3	529	-PPQIP--
At2g44830	AGC1-3	710	-PPEVPRQ
At5g40030	AGC1-4	456	-PPEIP--
At1g16440	AGC1-6/RSH3	449	-PPHLP--
At1g79250	AGC1-7	493	-PPHVP--
At3g12690	AGC1-5	522	-PPHIP--
At5g03640	AGC1-8	891	-PPELP--
At2g36350	AGC1-9	911	-PPELP--
At3g52890	KIPK	892	-PPELP--
At2g34650	PID	407	-PPEIP--
At2g2670	AGC3-4	478	-PPWVP--
At1g53700	WAG1	413	-PPE-IRG
At3g14370	WAG2	409	-PPEEVRG
At3g45780	Phot1	965	-PPELE--
At5g58140	Phot2	877	-PPPLD--
At4g13000	AGC2-2	335	RPPYIPAP
At3g25250	AGC2-1/OXI1	342	RPPYIPAP
At1g51170	AGC2-3/UCN	353	RPPFIPLR
At3g20830	AGC2-4/UCNL	354	RPPFIPLR
At4g14350	NDR-1	430	KAAFIPQV
At1g03920	NDR-2	447	EAAFIPEV
At3g23310	NDR-3	433	KAAFIPQV
At2g19400	NDR-4	526	DAAFKPVV
At2g20470	NDR-5	533	DAAFVPEV
At4g33080	NDR-6	414	EAAYKPEV
At1g30640	NDR-7	435	NAPYIPQV
At5g09890	NDR-8	416	EAAYRPIV
At5g62310	IRE	1055	KAMFVPSA
At1g48490	IRE-3	1123	KAAFVPDS
At1g45160	IRE-4	967	KAAFVPQP
At3g17850	IRE-H1	1183	KAAFVPAS
At3g08730	S6K1	403	MPSFKPEV
At3g08720	S6K2	409	QPSFKPAV
At5g04510	PDK1-1	324	PPKLAPDP
At3g10540	PDK1-2	325	PPKLAPDP

M.polymorpha

Mapoly0030s0017.1		501	TPPDIPKP
Mapoly0008s0088.1		482	LPPQIPRP
Mapoly0133s0008.1	Phot	1080	TPPPLETP
Mapoly0060s0068.1		379	SRTPVVPP
Mapoly0015s0182.1		305	QPPPLCLP
Mapoly0070s0030.1		457	QLKPEFKP
Mapoly0084s0015.1		1035	KAAFVPSP
Mapoly0019s0183.1		453	EAAFKPEV
Mapoly0001s0303.1		444	EAAFKPEV

b

C. elegans	Akt1b	469	EPPYKPNV
D. Melanogaster	Akt1	456	PPPFKPQV
M. musculus	Akt1	422	SPPFKPQV
H. sapiens	Akt1	422	SPPFKPQV
C. reinhardtii	Phot	725	QPPYVPRR
M. polymorpha	Phot	1080	TPPPLETP
A. thaliana	Phot1	964	NPPELETP
A. thaliana	Phot2	876	SPPPLDAP
A. capillus-veneris	Phot1	1055	RPPTFDAP
A. capillus-veneris	Phot2	980	VPPPLEAP
O. sativa	Phot1	893	APPKLEIP
O. sativa	Phot2	875	SPPELDVP

Figure S4. Polyproline within the C-terminal region of AGC kinases. a, Multiple alignment of the C-terminal sequences of all AGC kinases from *Arabidopsis thaliana* and *Marchantia polymorpha*. Prolines are highlighted in red. **b**, Alignment of the PxxP and PP motifs. Akt proteins are AGC kinases from animals: Akt1b (*Caenorhabditis elegans*), Akt1 (*Drosophila melanogaster*), Akt1 (*Mus musculus*), Akt1 (*Homo sapiens*); Phot (*Chlamydomonas reinhardtii*), Phot (*Marchantia polymorpha*), Phot1 and Phot2 (*Arabidopsis thaliana*), Phot1 and Phot2 (*Adiantum capillus-veneris*), and Phot1 and Phot2 (*Oryza sativa*). Prolines are highlighted in red.

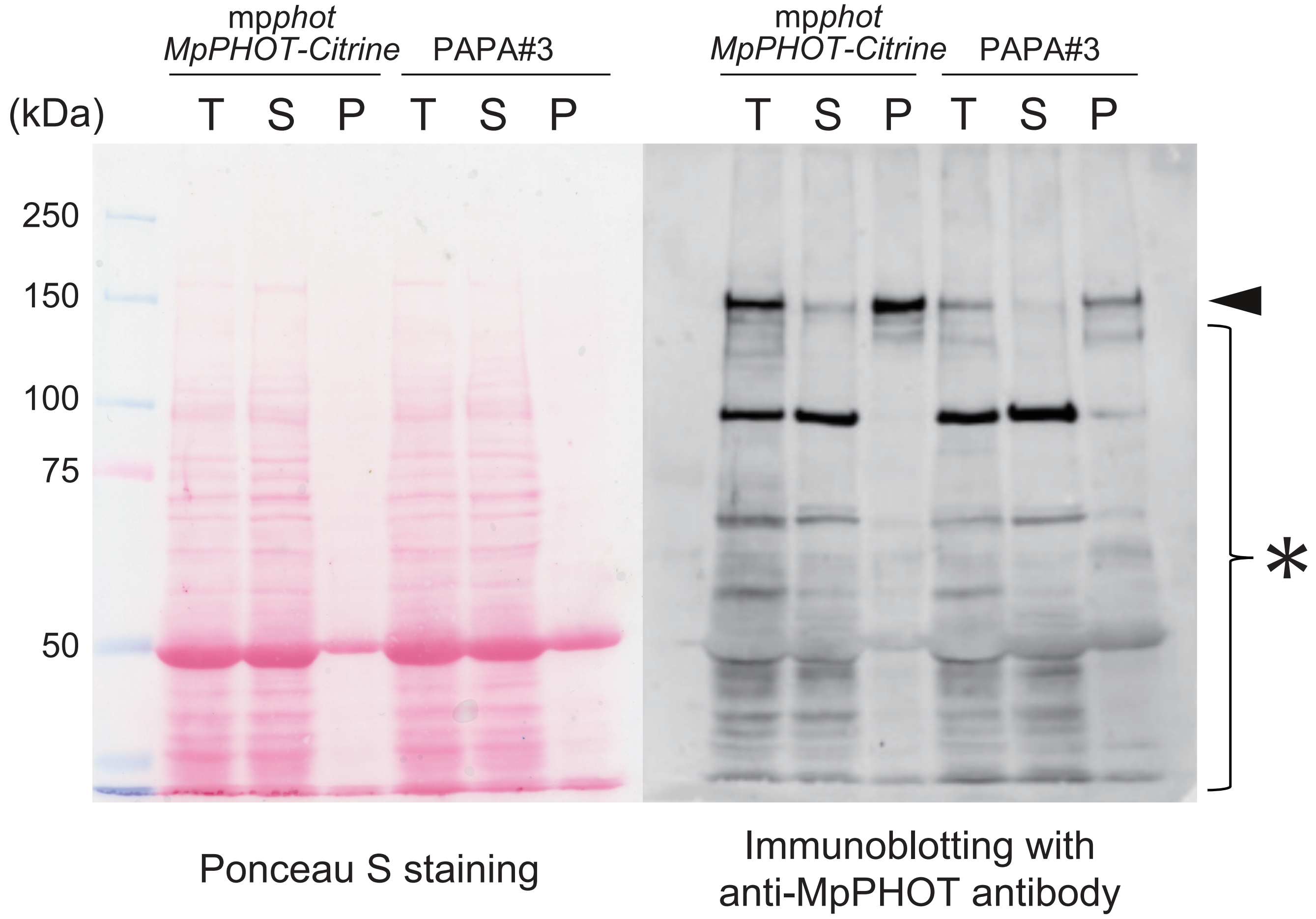

Figure S5. Biochemical fractionation of protein extracts from mp*phot* *MpPHOT-Citrine* and mp*phot* *MpPHOT-PAPA-Citrine*. Total protein (T) was extracted from 2-week-old transgenic mp*phot MpPHOT-Citrine* and mp*phot MpPHOT-PAPA-Citrine* (PAPA line #3) gemmaling cells and fractionated into soluble (S) and pellet (P) fractions. The total protein, soluble, and pellet fractions were subjected to SDS-PAGE and immunoblot analysis with an anti-Mpphot polyclonal antibody (right panel) (4). Ponceau staining of Rubisco large subunit (RbcL) is shown as the loading control (left panel) (46). Arrowhead indicates Mpphot-Citrine and Mpphot-PAPA-Citrine. Asterisk indicates non-specific signal.

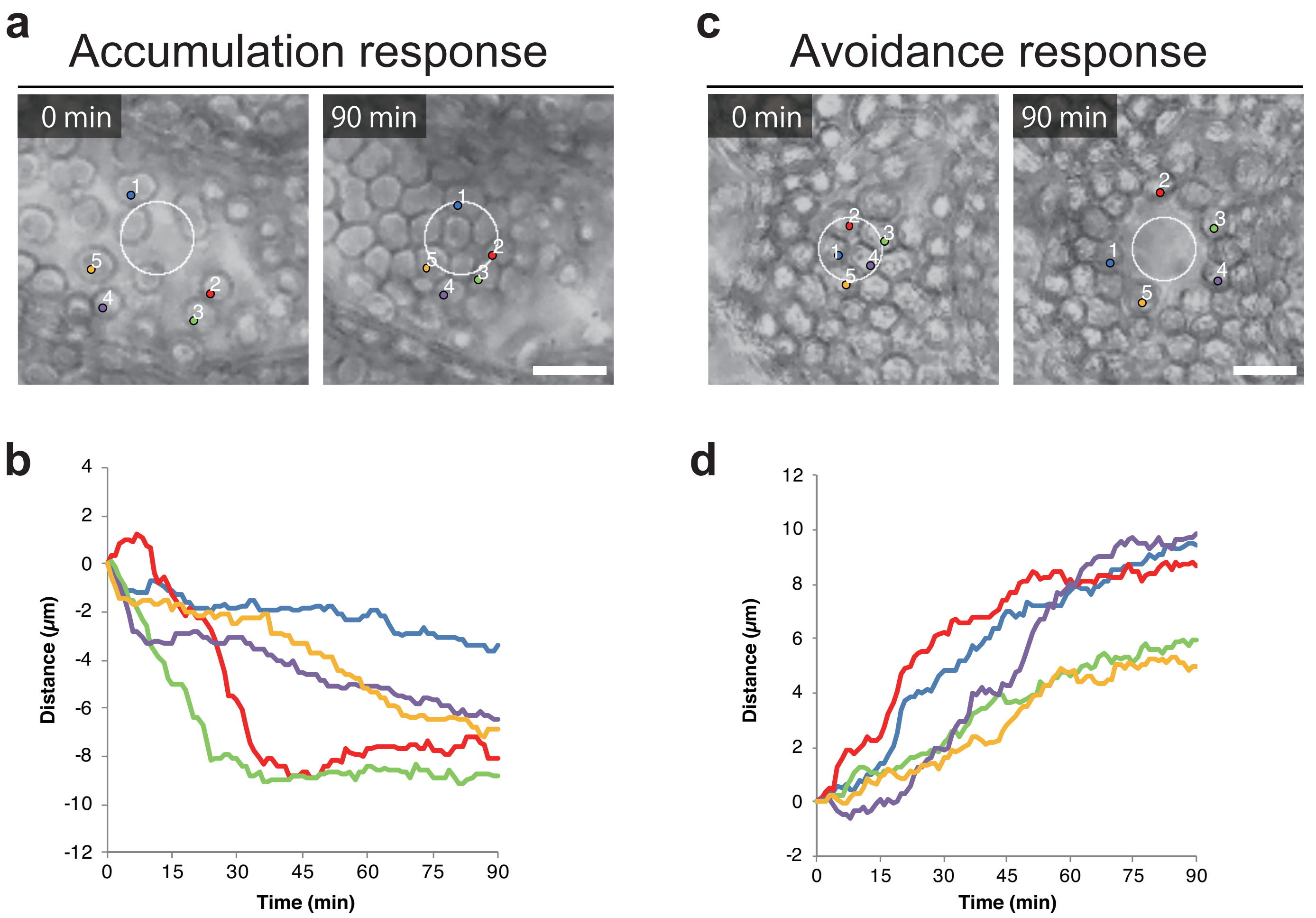

Figure S6. The accumulation and avoidance responses of chloroplasts in transgenic mp*phot Myri-Mpphot-PAPA-Citrine M. polymorpha* lines. **a**, The accumulation response in mp*phot Myri-MpPHOT-PAPA-Citrine* (Myri-PAPA line #3). The large white circle indicates the area irradiated with a weak blue-light microbeam (10 W m^{-2}); the small filled circles indicate the position of randomly selected chloroplasts. Left (0 min), before irradiation; right (90 min), after microbeam irradiation. Scale bar, 10 μm. **b**, Time-dependent change in distance between the center of the microbeam and the position of selected chloroplasts shown in **a** from the initial position (0 on the *y*-axis) to the end of the data capture (90 min) under microbeam-mediated irradiation. **c**, The avoidance response in Myri-PAPA line #3. The large white circle indicates the area irradiated with a strong blue-light microbeam (100 W m^{-2}); the small filled circles indicate the position of randomly selected chloroplasts. Left (0 min) and right (90 min) panels indicate the positions of chloroplasts before and after microbeam irradiation, respectively. Scale bar, 10 μm. **d**, Time-dependent change in distance between the center of the microbeam and the position of selected chloroplasts shown in **c** from the initial position (0 on the *y*-axis) to the end of the data capture (90 min) under microbeam-mediated irradiation.

Table S1. The pDONR plasmids consructed in this study.

pDONR plasmids	DNA templates	Primer names	Primer sequences (5' to 3' direction)
pDONR207-MpPHOT-ΔC19	pDONR207-MpPHOT[*1]	MpPHOT-attB1	GGGGACAAGTTTGTACAAAAAAGCAGGCTTCATGATGCCCTCCACGGAT
		MpPHOT-3270-3288-attB2	GGGGACCACTTTGTACAAGAAAGCTGGGTCGTCCGCTTCTTTGCCGATT
pDONR207-MpPHOT-ΔC38	pDONR207-MpPHOT[*1]	MpPHOT-attB1	GGGGACAAGTTTGTACAAAAAAGCAGGCTTCATGATGCCCTCCACGGAT
		MpPHOT-deltaC38-attB2	GGGGACCACTTTGTACAAGAAAGCTGGGTCGCGGATGAGAGGCCAATTG
pDONR207-MpPHOT-P1A	pDONR207-MpPHOT[*1]	MpPHOT(P1081A)-F	CATCCGCTGCATGACTGCCCCACCGCTAGAAACAC
		MpPHOT(P1081A)-R	GTGTTTCTAGCGGTGGGGCAGTCATGCAGCGGATG
pDONR207-MpPHOT-P2A	pDONR207-MpPHOT[*1]	MpPHOT(P1082A)-F	CCGCTGCATGACTCCTGCCCCGCTAGAAACACCAA
		MpPHOT(P1082A)-R	TTGGTGTTTCTAGCGGGGCAGGAGTCATGCAGCGG
pDONR207-MpPHOT-PAPA	pDONR207-MpPHOT-P1A	MpPHOT(P1081A/P1082A)-F	CGCTGCATGACTGCCGCCCCGCTAGAAACACCA
		MpPHOT(P1081A/P1082A)-R	TGGTGTTTCTAGCGGGGCGGCAGTCATGCAGCG
pDONRZeo-Myri-MpPHOT-PAPA	pDONR207-MpPHOT-PAPA	Myri-MpPHOT-attB1	GGGGACAAGTTTGTACAAAAAAGCAGGCTTCatgggaatctgcatgtcccgcATGATGCCCTCCACGGAT[*2]
		MpPHOT-attB2	GGGGACCACTTTGTACAAGAAAGCTGGGTCATATTCATCAAATGAGGCG

[*1]pDONR207-MpPHOT was previously reported (Kodama 2016 PLOS ONE 11:e0152484)

[*2]Lower case letters indicate a sequence encoding a myristoylation signal [MGICMSR].
